# The *IL6* Gene Promoter SNP and Plasma IL-6 in Response to Diet Intervention

**DOI:** 10.3390/nu9060552

**Published:** 2017-05-27

**Authors:** Brinda K. Rana, Shirley W. Flatt, Dennis D. Health, Bilge Pakiz, Elizabeth L. Quintana, Loki Natarajan, Cheryl L. Rock

**Affiliations:** 1 Department of Psychiatry, School of Medicine, University of California, San Diego, La Jolla, CA 92093-0738, USA; 2 Department of Family Medicine and Public Health, School of Medicine, University of California, San Diego, La Jolla, CA 92093-0901, USA; sflatt@ucsd.edu (S.W.F.); dheath@ucsd.edu (D.D.H.); bpakiz@ucsd.edu (B.P.); Elquintana@ucsd.edu (E.L.Q.); lnatarajan@ucsd.edu (L.N.); clrock@ucsd.edu (C.L.R.)

**Keywords:** IL-6, rs1800795, diet intervention, BMI, walnut

## Abstract

We recently reported that interleukin-6 (IL-6), an inflammatory marker associated with breast pathology and the development of breast cancer, decreases with diet intervention and weight loss in both insulin-sensitive and insulin-resistant obese women. Here, we tested whether an individual’s genotype at an *IL6* SNP, rs1800795, which has previously been associated with circulating IL-6 levels, contributes to changes in IL-6 levels or modifies the effect of diet composition on IL-6 in these women. We genotyped rs1800795 in overweight/obese women (*N* = 242) who were randomly assigned to a lower fat (20% energy), higher carbohydrate (65% energy) diet; a lower carbohydrate (45% energy), higher fat (35% energy) diet; or a walnut-rich (18% energy), higher fat (35% energy), lower carbohydrate (45% energy) diet in a 1-year weight loss intervention study of obesity-related biomarkers for breast cancer incidence and mortality. Plasma IL-6 levels were measured at baseline, 6 and 12 months. At baseline, individuals with a CC genotype had significantly lower IL-6 levels than individuals with either a GC or GG genotype (*p* < 0.03; 2.72 pg/mL vs. 2.04 pg/mL), but this result was not significant when body mass index (BMI) was accounted for; the CC genotype group had lower BMI (*p* = 0.03; 32.5 kg/m^2^ vs. 33.6 kg/m^2^). We did not observe a 2-way interaction of time*rs1800795 genotype or diet*rs1800795 genotype. Our findings provide evidence that rs1800795 is associated with IL-6 levels, but do not support a differential interaction effect of rs1800795 and diet composition or time on changes in circulating IL-6 levels. Diet intervention and weight loss are an important strategy for reducing plasma IL-6, a risk factor of breast cancer in women, regardless of their rs1800795 genotype.

## 1. Introduction

Elevated interleukin-6 (IL-6) has been consistently associated with breast pathology and the development of breast cancer [1]. The *IL6* rs1800795 SNP (-174G>C) is a focus of genetic studies on breast cancer risk because of its association with circulating IL-6 levels [2]. A study of 624 primary breast cancer patients in Sweden revealed that C carriers had a higher risk of early events than GG carriers [3]. However, the body of genetic studies have been inconclusive. A recent meta-analysis (12 studies; 10,137 breast cancer cases, 15,566 controls) was unable to establish an association between *IL6* genotypes and breast cancer risk [4]. 

We recently reported that diet intervention and weight loss are associated with decreased IL-6 in both insulin-sensitive and insulin-resistant obese women [5]. We follow-up with the question: does the rs1800795 genotype interact with the type of diet composition or time to affect IL-6? If so, rs1800795 may be used to personalize a weight reduction regimen to reduce breast cancer risk. 

## 2. Materials and Methods 

### 2.1. Subjects

Participants (*N* = 242) were from a behavioral weight loss intervention trial in San Diego, CA [5] which randomized 245 overweight and obese women from a screened sample of 1559 women. Inclusion criteria were: female, ≥21 years old; body mass index (BMI) between 27 and 40 kg/m^2^; willing and able to participate in clinic visits, group sessions, and telephone and internet communications; able to provide data through questionnaires and telephone; willing to maintain contact with investigators for 12 months; willing to allow blood collections; no known allergy to tree nuts; and capable of performing a simple test for assessing cardiopulmonary fitness. Exclusion criteria were any of the following: inability to participate in physical activity due to severe disability; history or presence of a comorbid diseases where diet modification and increased physical activity may be contraindicated; self-reported pregnancy or breastfeeding or planning a pregnancy within the next year; currently involved in another diet intervention study or weight loss program; and having a history or presence of a significant psychiatric disorder or any condition that would interfere with participation in trial. The University of California, San Diego institutional review board approved the study protocol, and all participants provided written informed consent (Clinical Trial Registration: NCT01424007 [6]. Prior to enrollment, women were screened for diabetes and considered ineligible with a fasting blood glucose ≥125 mg/dL. 

Enrolled participants were randomly assigned to one of the three study arms: lower fat (20% energy), higher carbohydrate (65% energy) diet; higher fat (35% energy), lower carbohydrate (45% energy) diet; or walnut-rich (18% energy), higher fat (35% energy), lower carbohydrate (45% energy) diet. All diet prescriptions limited saturated fat, so guidance for the higher fat diet emphasized lean meats and reduced-fat dairy foods, while encouraging monounsaturated fat as a major, but not sole source of fat in the diet. In all diets, prescribed protein intake exceeded recommended levels although lower fat diet had slightly lower levels compared to the other two diets. The randomization was stratified by menopausal status (older/younger than 55 years as a proxy) and by insulin resistance status (insulin sensitive or insulin resistant) with a homeostasis model assessment (HOMA) value of >3.0 indicative of insulin resistance. Fasting glucose and insulin were measured at the screening clinic visit to calculate HOMA for the baseline characterization of insulin resistance status. Data collection, anthropometric measurements and blood sample collection were conducted at clinic visits at baseline and 6 and 12 months. 

Following randomization, participants were provided a detailed diet prescription and sample meal plans in an individual counseling session with a dietitian, with follow-up at regular intervals by telephone or email to provide additional support and reinforce adherence. The overall goal of the dietary guidance was to promote a reduction in energy intake, aiming for a 500–1000 kcal/day deficit relative to expenditure, according to the individualized prescribed diet plan (1200, 1500 or 1800 kcal/day). Specific instructions for the lower fat diet were to choose lean protein sources and reduced-fat dairy foods and to emphasize vegetables, fruit, and whole grains as healthy high-carbohydrate choices. Participants assigned to the lower carbohydrate diet were educated about higher vs. lower carbohydrate choices, as well as lean protein sources, and were instructed to achieve a high monounsaturated fat intake, and examples and recipes were discussed. 

Participants assigned to the walnut-rich study group were also educated about higher vs. lower carbohydrate choices and lean protein sources. They were also instructed to consume an average of 42 g (1.5 oz) walnuts per day, within their energy-reduced diet plan, and were provided meal and snack suggestions and recipes to facilitate adherence. Walnuts were distributed to participants assigned to that group approximately every two weeks and they were instructed to record walnut consumption on a simple form. Diet prescriptions for participants assigned to the other two study groups excluded nuts. 

All participants also were encouraged to use Web-based tracking programs that guide dietary intake toward the prescribed macronutrient distribution, conduct self-monitoring, and were provided both group-based behavioral weight loss intervention and one-on-one counseling on diet and activity as previously described [7].

### 2.2. Assays

We measured plasma IL-6 at baseline, 6 and 12 months using solid phase quantitative sandwich ELISA (R & D Systems, Inc., Minneapolis, MN, USA) with inter-assay Coefficients of Variability of 9%.

DNA was extracted from blood using the QIAamp DNA Blood Mini Kit (Qiagen, Hilden, Germany). Rs1800795 was genotyped using iPLEX Gold chemistry on a MassARRAY^®^ System (Agena Bioscience, San Diego, CA, USA) at the Roswell Park Cancer Institute Genomics Shared Resource.

### 2.3. Statistical Analysis

Of the 242 participants genotyped, 192 completed the study, and one with IL-6 values higher than 100 pg/mL was excluded from the analysis. Two-sample chi-square and *t*-tests were used to compare demographics, baseline IL-6 and BMI between genotype groups. Mixed effect models (MEM) were used to model associations between genotype and longitudinal IL-6 levels, and likelihood ratio tests were used to test interaction effects of genotype with diet or time. 

With a total sample size of 234 subjects, our study had 80% power to detect a weight loss difference of 3.8 kg and a between group biomarker (e.g., IL-6) effect-size of 0.89 between two diet arms in the insulin-sensitive or insulin-resistant subjects. As secondary analysis, our study explored whether biomarker changes between diet arms differed according to *IL6* genotype. We also conducted post-hoc power calculations for the main genotype effect on baseline IL-6 levels and the genotype*diet interaction on changes in IL*-*6, based on 2-sided tests with significance level set to 0.05. With 242 participants (18% CC genotype), we have 80% power to detect a mean difference of 0.68 pg/mL in the CC versus (GG or GC) groups assuming distributions similar to those observed in our study based on a 2-sided 2-sample *t*-test. For the interaction, we have 15% power to detect the observed 0.07 effect-size for changes in IL6 changes based on a F-test. Conversely, with the study sample size of 242, we have 80% power to detect a 0.2 interaction effect-size. Thus, for this exploratory analysis, our study had sufficient power to detect the observed differences in baseline IL6 levels by genotype, but not for genotype*diet effects on IL-6 changes. 

## 3. Results

Rs1800795 genotypes are summarized in Table 1: 42% GG, 40% GC, and 18% CC. The CC genotype was not present in the 13 African American (AA) women (seven GC, six GG). The SNP was in Hardy–Weinberg equilibrium (*χ*^2^ = 3.15) in the non-AA population. IL-6 and BMI at baseline were lower in participants with the CC genotype compared to carriers of the G allele.

In the mixed model, the CC group had lower IL6 compared to the GC/GG groups across time-points (*p* = 0.06, Model 1, Table 2), but this effect was attenuated with adjustment for BMI (Model 2, Table 2). Excluding AAs did not change results, so we report analyses with AAs included. The genotype*time interaction added to Model 1 was not significant, indicating that changes in marker levels were not different by genotype. Specifically, mean (95% CI) change in IL-6 levels between baseline and at 6 months for G allele carriers was −0.44 (−0.68, −0.20) compared to −0.44 (−0.96, +0.08) pg/mL for the CC group, and at 12 months, −0.99 (−1.24, −0.74) for G carriers versus −0.65 (−1.19, −0.11) for the CC group. Genotype did not modify the diet effect on longitudinal IL-6 levels (genotype and diet model vs. genotype, diet, genotype*diet model, likelihood ratio test *p* = 0.71).

## 4. Discussion and Conclusions

We did not observe a significant effect of the rs1800795 genotype on longitudinal plasma IL-6 levels or an interaction effect with time or diet composition in obese women undergoing a 12-month dietary intervention to promote weight loss. Our results are consistent with a dietary intervention study (314 men, 407 women) in which the C allele was associated with higher serum IL-6 in men, but not in women, and no genotype*diet interaction was found [8]. In our study, BMI was lower in the CC group at baseline and remained lower at 6 and 12 months, possibly confounding the results. Consistent with our previous report, BMI as well as time is driving IL-6 levels, not rs1800795 genotype [5].

A limitation to our study is that our cohort was not underpowered to identify genotype*diet interaction effects on IL-6 changes. However, with a sample size of 242, we were powered to detect changes in baseline IL-6 levels by genotype. The relationship between IL-6, breast cancer risk factors, obesity measures, and rs1800795 is complex and previous genetic studies have been conflicting [2,9]. These conflicts may be due to the role of other *IL6* SNPs and resolved by applying haplotype-based strategies which include other *IL6* SNPs, but such studies require larger cohorts that are difficult to obtain for a longitudinal intervention study such as ours.

We conclude that, as shown in our previous report, diet intervention and weight loss is an important strategy for reducing plasma IL-6, a risk factor of breast cancer in women, but the *IL6* rs1800795 genotype does not interact with the diet type or time to affect IL-6 levels in our cohort.

## Figures and Tables

**Table 1:** Baseline characteristics in 242 participants.

	rs1800795 Genotype		
Characteristics	GG or GC *N* (%)	CC *N* (%)	*p* ^b^
Race/Ethnicity			0.17
White	142(80.2)	35 (19.8)	
African American	13 (100)	0	
Asian American	4 (100)	0	
Hispanic	33 (82.5)	7 (17.5)	
Mixed/Other	7 (87.5)	1 (12.5)	
IL-6 pg/mL ^a^	2.72 (0.15)	2.04 (0.21)	0.01
IL-6 pg/mL non-African Americans ^a^	2.61 (0.15)	2.04 (0.21)	0.03
BMI (kg/m^2^) ^a^	33.6 (0.2)	32.5 (0.4)	0.03
BMI (kg/m^2^) non-African Americans ^a^	33.5 (0.2)	32.5 (0.4) ^c^	0.06

^a^ Mean (S.E.M); ^b^ *p* value from Chi-square or 2-sample *t*-tests; ^c^ There were no African Americans in the CC group. IL-6, Interleukin-6; BMI, Body Mass Index.

**Table 2:** Associations between IL-6 level and genotype.

		Model 1		Model 2	
		Coefficient (95% CI)	*p* ^a^	Coefficient (95% CI)	*p* ^a^
GENOTYPE	GG or GC (Reference)				
	CC genotype	−0.55 ± 0.57	0.06	−0.42 ± 0.55	0.14
TIME POINT	Baseline (Reference)				
	6 months	−0.44 ± 0.22	<001	−0.19 ± 0.25	0.13
	12 months	−0.93 ± 0.23	<001	−0.66 ± 0.26	<001
	Body Mass Index			0.10 ± 0.05	<001

^a^ Mixed effect model on *N* = 241 excluding one participant with plasma IL-6 >100 pg/mL. Coefficients were derived from Type III Sum of Squares. Model 1: IL-6 Level = Genotype + Time point + Random Intercept. Model 2: IL-6 Level = Genotype + Time point + BMI+ Random Intercept.
